# Risk Analysis of Prostate Cancer Treatments in Promoting Metabolic Syndrome Development and the Influence of Increased Metabolic Syndrome on Prostate Cancer Therapeutic Outcome

**DOI:** 10.1007/s12672-018-0335-8

**Published:** 2018-06-09

**Authors:** Zongping Chen, Jichun Deng, Yong Yan, Min Li, Chanjuan Chen, Chao Chen, Sicong Zhao, Tao Song, Tong Liu, Xin Wen, Yuhong Yao

**Affiliations:** 1 Department of Urology, Beijing Shijitan Hospital, Capital Medical University, Beijing, 100038 China; 2 Department of Urology, Affiliated Hospital of Zunyi Medical College, Zunyi, 563000 Guizhou China

**Keywords:** Metabolism syndrome, Prostate cancer, C-reactive protein, Platelet distribution width

## Abstract

In clinical practice, few prostate cancer (PCa) patients are associated with metabolic syndrome (MetS), while few others acquire MetS during treatment. Whether the treatment of PCa increases the occurrence of MetS remains to be confirmed. This study reviewed the changes in MetS patients before and after PCa treatment to evaluate the effects of various treatment methods on MetS. We analyzed data of 1162 PCa patients, whether or not diagnosed with MetS, and changes in MetS patients after PCa treatment. Data of lower urinary tract symptoms, C-reactive protein (CRP), platelet distribution width (PDW), prostate-specific antigen (PSA), Gleason score, clinical stage, treatment methods, and progressive incidents were evaluated using logistic regression according to MetS diagnosis. The results showed significant differences in the prevalence of MetS before (17.38%) and after (23.67%) PCa treatment (*P* < 0.001). Bad diet, living habits, and prostate cancer treatment were considered as risk factors for MetS (OR = 1.731, 95%CI 1.367–2.193, *P* < 0.001). Radical prostatectomy (RP), androgen deprivation therapy including surgical castration and medical castration, iodine-125 seed brachytherapy (^125^I limited), and chemotherapy were independent risk factors of MetS. The MetS incidence rates after treatment in ADT+^125^I limited+chemotherapy compared to RP+TURP+EBRT were statistically significant at the corresponding risk grade (all *P* < 0.001). After treatment, the occurrence rates of progressive incidences were higher in MetS-PCa patients compared to non-MetS-PCa patients (all *P* < 0.001). So, the findings suggested that among PCa patients, multiple factors contribute to the occurrence of MetS, and PCa treatment is one among them. ADT+^125^I limited+chemotherapy may be the most influential treatment for MetS.

## Introduction

In clinical practice, few prostate cancer (PCa) patients were generally combined with metabolic syndrome (MetS), while few others acquire MetS during the treatment. Furthermore, the presence of MetS affects the prognosis of PCa and the selection of treatment. Whether the treatment of PCa itself increases MetS remains to be confirmed.

PCa is the second most common cancer among males over 60 years of age, next to lung cancer. With the widespread and increased detection of serum prostate-specific antigen (PSA) levels and digital rectal examination (DRE), the incidence of PCa has been increased dramatically. Many treatment options have been used clinically for the treatment of PCa, including radical prostatectomy (RP), radiation therapy, androgen deprivation therapy (ADT), emasculation, chemotherapy, palliative transurethral resection of prostate (TURP), and active surveillance. Mortality and survival rates in treated population vary due to disparities in the treatments or responses and prognostic factors, such as chronic comorbidities [1].

MetS is described as a complex medical disorder associated with a constellation of metabolic abnormalities. According to the National Cholesterol Education Program’s Adult Treatment Panel III (NCEP-ATPIII), the most widely accepted definition of MetS is that patients including at least three of the following factors: abdominal obesity (waist circumference > 90 cm in men or > 85 cm in women), hypertriglyceridemia (triglycerides > 150 mg/dl), low high-density lipoprotein cholesterol (HDL-C < 40 mg/dl in men and < 50 mg/dl in women), elevated blood pressure (> 130/85 mmHg), and a high fasting plasma glucose (FPG)level (> 110 mg/dl) [2]. MetS has become a major public health problem in many countries due to its increasing prevalence. In fact, MetS is one of the most common comorbidity observed in a majority of PCa patients during the development of disease course.

Recently, increasing evidence support the hypothesis that different metabolic factors and MetS may be involved in the development and progression of PCa [3]. Even though conflicting results have been shown in several studies [4, 5], many data supported the existence of a possible association between PCa and MetS. MetS subsequently impacted on PCa incidence, aggressiveness, and outcomes [6, 7]. The biological mechanisms explaining these findings remain unknown. MetS is associated with a pro-inflammatory state [8–10], which in turn is related to the risk of PCa [8, 10]. Despite their beneficial effects on survival, various therapeutic methods, especially ADT, may also induce numerous adverse effects that overlap with MetS and remains poorly understood [11–16].

Hence, the present study was carried out retrospectively in a multicenter with different cohorts of PCa patients to investigate the association between various treatments and MetS, influence on the outcomes, and their accompanied chronic inflammatory state.

## Patients and Methods

### Patients

This study was a retrospective cohort study. A consecutive series of data covering 1162 PCa patients during the period of January 2006 to December 2015 were collected from two medical centers (Beijing Shijitan Hospital, Capital Medical University, and Affiliated Hospital of Zunyi Medical College in China). Inclusion criteria were as follows: clinical records of all PCa patients’ (hospitalizations during January 1, 2006, to December 31, 2015) should be complete and accurate, and the follow-up visits lasted for 1–10 years. Exclusion criteria were as follows: (i) non-prostate-cancer (non-PCa) patients; (ii) incomplete clinical records; and (iii) PCa patients who are willing to terminate the treatment or refused for follow-up visits. The Institutional Review Board of the Beijing Shijitan Hospital approved the present study in September 2014.

### Clinical Data Extraction

Available parameters included C-reactive protein (CRP), platelet distribution width (PDW), PSA, pathological Gleason score, clinical stages of primary tumor, lower urinary tract symptoms (including storage and voiding symptoms), the treatments, dwelling environment, living habits (including smoking, drinking, diets, and sports), education background, the components of MetS including waist circumference ≥ 90 cm, triglycerides ≥ 1.70 mmol/l, HDL-C < 1.04 mmol/l, blood pressure ≥ 130/85 mmHg, and FPG ≥ 6.1 mmol/l or 2-h postprandial blood glucose (2hPG) ≥ 7.8 mmol/l), as well as the progressive incidents of PCa (defined as significant PSA increase, tumor recurrence, distant metastasis, and death in the present context). The subjects were evaluated using different risk grades of PCa (low-risk, intermediate-risk, and high-risk) according to the National Comprehensive Cancer Network (NCCN) criteria [17]. The presence of MetS was defined according to the updated guidelines of NCEP-ATP III [2].

### Diagnosis Standards

MetS was diagnosed using the 2005 NCEP-ATP III criteria for Asian Americans [2]. Diagnostic points of PCa patients included during the first visit and treatment. The modified NCEP-ATP III has defined MetS as the simultaneous occurrence of at least three of the following five risk factors: (1) waist circumference ≥ 90 cm, (2) triglycerides ≥ 1.70 mmol/l or drug treatment for elevated triglycerides, (3) HDL-C < 1.04 mmol/l or drug treatment for reduced HDL-C, (4) blood pressure ≥ 130/85 mmHg or antihypertensive drug treatment with a history of hypertension, and (5) FPG ≥ 6.1 mmol/l or 2hPG ≥ 7.8 mmol/l or drug treatments for elevated glucose. We stratified the subjects into six metabolic components according to their met criteria (0, 1, 2, 3, 4, and 5). MetS is defined as the inclusion of 3–5 metabolic components of abnormality, and non-MetS included 0–2 metabolic components [18]. PCa was diagnosed using prostate biopsy. Clinical staging of primary tumor of PCa was done according to the 2002 American Joint Committee on Cancer (AJCC) clinical Stage of prostate carcinoma. Progression of PCa was diagnosed with at least one of the following factors: presence of progressive PSA increase (PSA > 10 ng/ml), cancer recurrence, metastasis, and death.

### Follow-Up

The median follow-up time was 5.5 (range 1–10) years. All the patients were followed up through telephone, regular outpatient visits, and regular in-patient visits. Follow-up items through telephone consultation included health status and death, outpatient and hospitalization included general physical examination, blood routine analysis, blood biochemical examination, chest abdominal computed tomography (CT), and whole-body bone scanning.

### Statistical Analyses

Statistical analyses were performed using Statistical Package for the Social Science (SPSS Inc., Chicago, USA) version 18.0 for Windows (SPSS Inc., Chicago, IL, USA). Selected characteristics (including clinical data parameters described above are collected) were compared between MetS cases and non-MetS cases using chi-square test for categorical variables. For non-normally distributed factors on raw or log-transformed scales, rank-sum test was used to evaluate the differences in PSA, storage symptoms, voiding symptoms, CRP, and PDW. Multivariate logistic regression analyses adjusted for potential confounders (age, smoking status, drinking condition, diet information, physical exercise situation, dwelling environment, education background) were performed to assess the association of MetS with different treatments, PSA, Gleason scores, clinical stages, storage symptoms, voiding symptoms, CRP, and PDW. Multivariate-adjusted odds ratios (ORs) and 95% confidence intervals (CIs) were simultaneously estimated by logistic regression analyses. Kaplan-Meier curves through log-rank test and Cox regression model were used to assess the risk of survival between MetS cases and non-MetS cases. Differences were considered statistically significant when *P* values were < 0.05.

## Results

### Baseline Characteristics of PCa Patients During Their First Visit to a Doctor As Well As the Changes of MetS and Its Components Before and After Treatment

**Fig. 1:**
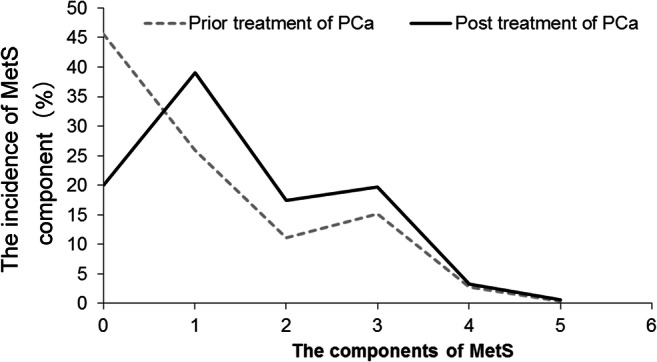
Comparison of component change of metabolic syndrome prior treatment with posttreatment of prostate cancer (*n* = 1162, %). MetS included 3–5 components to abnormality, and non-MetS included 0–2 components. *P* < 0.001 for comparison of prior treatment with posttreatment. *P* value was calculated using chi-square. *P* < 0.05 was considered statistically significant

Patient’s age ranged from 43 to 97, with an average age of 76.5. A total of 1162 PCa patients were enrolled on admission including 202 (17.38%) patients with MetS and 960 (82.62%) were without MetS at baseline (Table 1). After undergoing treatment, 155 MetS patients had no MetS. Meanwhile, 228 without MetS patients developed MetS during the follow-up period. These results showed an increased number of patients with MetS [17.38% (202/1162) vs 23.67% (275/1162)]. At the same time, the individuals without MetS showed a downward trend [82.62% (960/1162) vs 76.33% (887/1162)] before treatment vs posttreatment, and the difference was statistically significant (*P* < 0.001). The incidence of MetS was higher in patients with unhealthy living habits (smoking, drinking, high-carb high-fat diet, and lack of sports), living in cities, high academic qualifications, and increased age (all *P* < 0.001). PSA levels, storage symptoms, voiding symptoms, CRP, and PDW were higher in MetS group than in non-MetS group (*P* = 0.007, < 0.001, = 0.048, = 0.002, = 0.005, respectively). The percentage of PSA levels (< 10, 10–20, > 20 ng/ml), Gleason scores (≤ 6, 7, ≥ 8) and clinical stages (≤ T2a, T2b, ≥ T2c) in MetS group were different compared to non-MetS group (*P* < 0.001, = 0.045, < 0.001, respectively). Furthermore, as shown in Fig. 1, it was noteworthy that the change of MetS component abnormalities was more obvious after undergoing cancer treatments. The total proportion of patients with one or more component abnormalities of MetS was higher in the treated patients than in patients before treatment (80.08 vs 55.42%, *P* < 0.001). The increase of one component abnormality of MetS was considered to be significant, followed by those with two and three component abnormalities, while increase to four and five component abnormalities remained to be the lowest. In a word, differences existed in the incidence of MetS before and after cancer treatment, and the general trend was that the incidence of MetS posttreatment was higher than before treatment, which was independently associated with various treatments (OR = 1.731, 95%CI 1.367–2.193, *P* < 0.001) through multivariate logistic regression analysis (Table 1) after adjusting for age (OR = 1.042, 95%CI 1.028–1.056, *P* < 0.001), smoking (OR = 0.299, 95%CI 0.197–0.456, *P* < 0.001), drinking (OR = 2.501, 95%CI 1.767–3.541, *P* < 0.001), high-fat high-calorie diet (OR = 2.771, 95%CI 2.099–3.657, *P* < 0.001), lack of physical exercise (OR = 4.827, 95%CI 3.521–6.617, *P* < 0.001), PSA (OR = 1.000, 95%CI 0.999–1.001, *P* = 0.046), Gleason scores (OR = 1.658, 95%CI 1.362–2.018, *P* < 0.001), clinical stages (OR = 1.256, 95%CI 1.025–1.538, *P* = 0.028), storage symptoms (OR = 1.060, 95%CI 1.000–1.123, *P* = 0.045), voiding symptoms (OR = 1.227, 95%CI 1.110–1.358, *P* < 0.001). The dwelling environment (OR = 0.915, 95%CI 0.663–1.263, *P* = 0.587), and education background (OR = 0.911, 95%CI 0.785–1.057, *P* = 0.220) showed no association through multivariate logistic regression analysis (Table 1).

**Table 1:** The association of clinical characteristics of prostate cancer patients according to the status combined with and without metabolic syndrome

Variables	Overall (*n* = 1162)	MetS	Non-MetS	*P* value^a^	OR	*P* value^b^	95%CI
Undergoing treatment (%)				< 0.001*	1.731	*< 0.001*	1.367–2.193
Prior	1162 (100)	202/1162 (17.38)	960/1162 (82.62)				
Post	1162 (100)	275/1162 (23.67)	887/1162 (76.33)				
Age (years, %)				< 0.001*	1.042	*< 0.001*	1.028–1.056
< 60	102 (8.8)	29 (2.5)	93 (6.3)				
60–70	146 (12.6)	46 (4.0)	100 (8.6)				
> 70	914 (78.6)	127 (10.9)	767 (67.8)				
Smoking status (%)				< 0.001*	0.299	*< 0.001*	0.197–0.456
Smoking	340 (29.3)	191 (16.4)	149 (12.9)				
No smoking	822 (70.7)	15 (1.3)	807 (69.4)				
Drinking condition (%)				< 0.001*	2.501	*< 0.001*	1.767–3.541
Drinking	343 (29.5)	195 (16.8)	148 (12.7)				
No drinking	819 (70.5)	7 (0.6)	812 (69.9)				
Diet information (%)				< 0.001*	2.771	*< 0.001*	2.099–3.657
High-fat high-calorie	379 (32.6)	202 (17.4)	177 (15.2)				
Regular	783 (67.4)	0 (0.0)	783 (67.4)				
Physical exercise situation (%)				< 0.001*	4.827	*< 0.001*	3.521–6.617
Keep exercising	863 (74.3)	8 (0.7)	855 (73.6)				
Lack of exercise	299 (25.7)	194 (16.7)	105 (9.0)				
Dwelling environment (%)				< 0.001*	0.915	0.587	0.663–1.263
Living in city	662 (57.0)	148 (12.7)	514 (44.3)				
Living in the country	500 (43.0)	55 (4.7)	445 (38.3)				
Education background (%)				< 0.001*	0.911	0.220	0.785–1.057
≤ 6 years	501 (43.12)	58 (5.00)	443 (38.12)				
7–12 years	386 (33.22)	64 (5.51)	322 (27.71)				
≥ 13 years	275 (23.67)	80 (6.88)	195 (16.78)				
PSA (ng/ml) total	99 (4~1145)	133 (4.6~1145)	91 (4~1087)	*0.007*†	1.000	*0.046*	0.999–1.001
%				< 0.001*	0.997	*< 0.001*	0.995–0.999
< 10 ng/ml	29 (2.5)	3 (0.3)	26 (2.2)				
10–20 ng/ml	80 (6.9)	53 (4.6)	27 (2.3)				
> 20 ng/ml	1053 (90.6)	144 (12.4)	909 (78.2)				
Gleason scores (%)				0.045*	1.658	*< 0.001*	1.362–2.018
≤ 6	150 (12.9)	17 (1.5)	133 (11.4)				
7	209 (18.0)	41 (3.5)	168 (14.6)				
≥ 8	803 (69.1)	145 (12.4)	658 (56.6)				
Clinical stages (%)				< 0.001*	1.256	*0.028*	1.025–1.538
≤ T2a	99 (8.5)	4 (0.3)	95 (8.2)				
T2b	283 (24.4)	9 (0.8)	274 (23.6)				
≥ T2c	780 (67.1)	189 (16.3)	591 (50.8)				
Storage symptom (median)	5 (2~9)	6 (3~9)	4 (2~7)	*< 0.001*†	1.060	*0.045*	1.000–1.123
Voiding symptom (median)	6 (2–15)	8 (3~15)	5 (2~10)	*0.048*†	1.227	*< 0.001*	1.110–1.358
CRP (median, mg/l)	7.3 (3.9~12.4)	8.6 (6.6~12.4)	5.7 (3.9~8.2)	*0.002*†	1.077	*0.022*	1.011–1.148
PDW (median, %)	14.8 (11.4~21.6)	17.2 (12.6~21.6)	13.6 (11.4~16.8)	*0.005*†	1.202	*< 0.001*	1.112–1.298

The italic represents statistical significance. MetS included 3–5 components to abnormality, and non-MetS included 0–2 components

*MetS* metabolic Syndrome, *PSA* prostate-specific antigen, *PDW* platelet distribution width, *CRP* C-reactive protein, *OR* odds ratio, *CI* confidence interval

^a^*P* values were calculated using *chi-square, †rank-sum test

^b^*P* values were calculated using multivariate logistic regression analysis

### Association of Risk Grades, Progressive Incidents of PCa with MetS As Well As the Change of Incidence of MetS Before and After Treatment by Different Treatment Strategies for PCa

As shown in Table 2, after adjusting for age, smoking status, drinking condition, diet information, physical exercise situation, dwelling environment, and education background, MetS was independently associated with increased advanced PSA (OR = 1.491, 95%CI 1.087–2.047, *P* = 0.013), recurrence (OR = 1.426, 95%CI 1.041–1.953, *P* = 0.027), and metastasis (OR = 1.703, 95%CI 1.222–2.375, *P* = 0.002). Multivariate logistic regression analysis showed no association of MetS with death (OR = 1.079, 95%CI 0.976–1.193, *P* = 0.137) and risk grades to low-risk (OR = 1.277, 95%CI 0.923–1.768, *P* = 0.140), intermediate-risk (OR = 0.867, 95%CI 0.695–1.081, *P* = 0.205), and high-risk (OR = 0.935, 95%CI 0.320–2.732, *P* = 0.902).

**Table 2:** The association of risk grades, progressive incidents of prostate cancer with metabolic syndrome

Variables	%	*P* value^c^	OR	*P* value^d^	95%CI
Low risk					
(+)^a^	13.64 (15/110)	*< 0.001*	1.277	0.140	0.923–1.768
(−)^b^	86.36 (95/110)				
Intermediate risk					
(+)^a^	19.53 (42/215)	*< 0.001*	0.867	0.205	0.695–1.081
(−)^b^	80.47 (173/215)				
High risk					
(+)^a^	17.32 (145/837)	*< 0.001*	0.935	0.902	0.320–2.732
(−)^b^	82.68 (692/837)				
Advanced PSA increased					
(+)^a^	20.53 (131/638)	*< 0.001*	1.491	*0.013*	1.087–2.047
(−)^b^	79.47 (507/638)				
Recurrence					
(+)^a^	21.27 (137/644)	*< 0.001*	1.426	*0.027*	1.041–1.953
(−)^b^	78.73 (507/644)				
Metastasis					
(+)^a^	18.45 (100/542)	*< 0.001*	1.703	*0.002*	1.222–2.375
(−)^b^	81.55 (442/542)				
Death					
(+)^a^	27.81 (84/302)	*< 0.001*	1.079	0.137	0.976–1.193
(−)^b^	72.19 (218/302)				

The italic represents statistical significance. Low risk: PSA < 10 ng/ml, Gleason score ≤ 6, clinical stage ≤ T2a; intermediate risk: PSA = 10–20 ng/ml, Gleason score = 7, clinical stage = T2b; high risk: PSA > 20, Gleason score ≥ 8, clinical stage ≥ T2c

*OR* odds ratio, *CI* confidence interval, *PSA* prostate-specific antigen

^a^MetS, included 3–5 components to abnormality

^b^Non-MetS, included 0–2 components

^c^Chi-square test

^d^Multivariate logistic regression analysis, adjusted for age, smoking status, drinking condition, diet information, physical exercise situation, and dwelling environment

As shown in Table 3, the incidence of MetS after various treatments for PCa showed different changes. The increased incidence of MetS was found after applying iodine-125 seed brachytherapy (^125^I limited), medical castration (MC), and chemotherapy, and the incidence rate was increased from 56/337(16.6%) vs 119/337(35.3%), 41/233(17.6%) vs 68/233(29.2%), and 12/102(11.8%) vs 24/102(23.5%). The difference in MetS incidence rates were statistically significant (*P* < 0.001, *P* = 0.003, and *P* = 0.026, respectively). However, the decreased MetS incidence was found after RP and surgical castration (SC), which included 20/93(21.5%) vs 9/93(9.7%) and 55/314(17.5%) vs 37/314(11.8%). The difference in the decreased MetS incidence rate was statistically significant (*P* = 0.025 and *P* = 0.042, respectively). The rates of MetS incidence remained unchanged after undergoing external beam radiation therapy [EBRT, 13/45(28.9%)] and TURP [5/38(13.2%)], and the differences showed no statistical significance (*P* = 0.092 and 0.086). Multivariate logistic regression analysis showed that MetS was significantly associated with ^125^I limited (OR = 2.147, 95%CI 1.484–3.108, *P* < 0.001), MC (OR = 1.930, 95%CI 1.243–2.996, *P =* 0.003), chemotherapy (OR = 2.308, 95%CI 1.083–4.917, *P =* 0.030), RP (OR = 0.391, 95%CI 0.168–0.912, *P* = 0.030), and SC (OR = 0.629, 95%CI 0.401–0.986, *P =* 0.043).

**Table 3:** Change in the incidence of metabolic syndrome before and after treatments by different options for prostate cancer

Variables	Number (prior/posttreatment)	% (prior/posttreatment)	*P* value^c^	OR	*P* value^d^	95%CI
RP						
(+)^a^	20/9	21.5/9.7	*0.025*	0.391	*0.030*	0.168–0.912
(−)^b^	73/84	78.5/90.3				
SC						
(+)^a^	55/37	17.5/11.8	*0.042*	0.629	*0.043*	0.401–0.986
(−)^b^	259/277	82.5/88.2				
MC						
(+)^a^	41/68	17.6/29.2	*0.003*	1.930	*0.003*	1.243–2.996
(−)^b^	192/165	82.4/70.8				
^125^I limited						
(+)^a^	56/119	16.6/35.3	*< 0.001*	2.147	*< 0.001*	1.484–3.108
(−)^b^	281/218	83.4/64.7				
EBRT						
(+)^a^	13/13	28.9/28.9	0.092	1.294	0.866	0.365–3.313
(−)^b^	32/32	71.1/71.1				
Chemotherapy						
(+)^a^	12/24	11.8/23.5	*0.026*	2.308	*0.030*	1.083–4.917
(−)^b^	90/78	88.2/76.5				
TURP						
(+)^a^	5/5	13.2/13.2	0.086	0.638	0.457	0.195–2.085
(−)^b^	33/33	86.8/86.8				

The boldface represents statistical significance

*RP* radical prostatectomy, *SC* surgical castration, *MC* medical castration, *EBRT* radiation therapy of prostate cancer, *TURP* transurethral resection of the prostate, *OR* odds ratio, *CI* confidence interval

^a^MetS, included 3–5 components to abnormality

^b^Non-MetS, included 0–2 components

^c^Chi-square test

^d^Multivariate logistic regression analysis, adjusted for age, smoking status, drinking condition, diet information, physical exercise situation, dwelling environment, PSA, Gleason scores, and clinical stages

### The Differences of Newly Developed MetS Patients After Treatments with RP+TURP+EBRT Group and ADT+^125^I Limited+Chemotherapy Group According to the Risk Grades of PCa and Compared the Incidence of PCa Progression in Low-/Intermediate-/High-Risk Grades Between MetS and Non-MetS Patients After Treatment

**Fig. 2:**
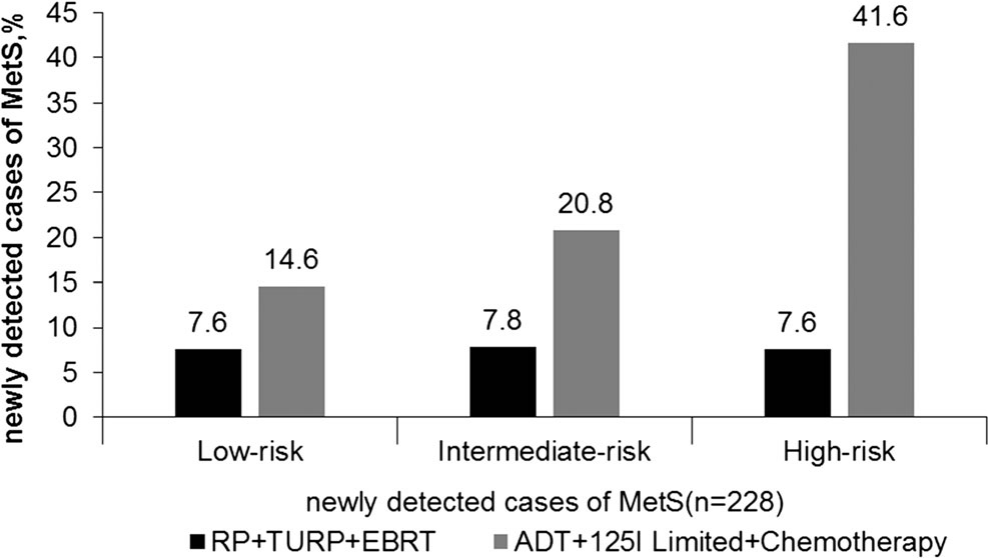
The differences of the newly developing cases of metabolic syndrome after the treatments of RP+TURP+EBRT group and ADT+^125^I limited+chemotherapy group according to risk grades of prostate cancer. RP radical prostatectomy, ADT androgen deprivation therapy, EBRT external radiation therapy of prostate cancer, TURP transurethral resection of the prostate. MetS including 3–5 components to abnormality. Low risk: PSA < 10 ng/ml, Gleason score ≤ 6, clinical stage ≤ T2a. Intermediate risk: PSA = 10–20 ng/ml, Gleason score = 7, clinical stage = T2b. High risk: PSA > 20, Gleason score ≥ 8, clinical stage ≥ T2c. *P* < 0.001 for comparing RP+TURP+EBRT with ADT+^125^I limited+chemotherapy in low-risk, intermediate-risk, and high-risk grades, respectively. *P* value was calculated using chi-square. *P* < 0.05 was considered statistically significant

We subjectively divided all the treatment methods into two groups, namely, ADT+^125^I limited+chemotherapy and RP+TURP+EBRT according to their effects on systemic metabolism that differed to analyze the MetS incidence rates. As shown in Fig. 2, the MetS incidence rates after treatment with ADT+^125^I limited+chemotherapy was 14.6% for low-risk group, 20.8% for intermediate-risk, and 41.6% for high-risk and with RP+ TURP+EBRT were 7.6% for low-risk, 7.8% for intermediate-risk, and 7.6% for high-risk. The differences of newly detected MetS (*n* = 228) incidence between the two treatment groups in the same risk group were very obvious and statistically significant (all *P* < 0.001).

**Fig. 3:**
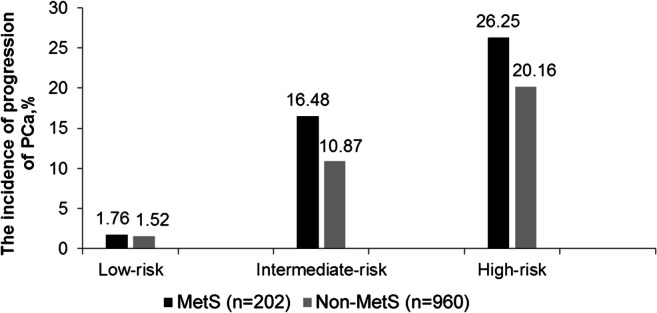
Comparison of the incidence of progression of PCa in low-/intermediate-/high-risk grades between MetS with non-MetS after treatment (%). MetS included 3–5 components to abnormality; non-MetS included 0–2 components. Low risk: PSA < 10 ng/ml, Gleason score ≤ 6, clinical stage ≤ T2a. Intermediate risk: PSA = 10-20 ng/ml, Gleason score = 7, clinical stage = T2b. High-risk: PSA > 20, Gleason score ≥ 8, clinical stage ≥ T2c. All *P* < 0.001 in low-risk, intermediate-risk, and high-risk grades for comparing MetS group with non-MetS group. *P* value was calculated using chi-square. *P* < 0.05 was considered statistically significant

The influence of MetS on tumor progression and risk development after treatment were analyzed between the two groups of patients with or without MetS. As shown in Fig. 3, after treatment, the risk of PCa progression was more remarkably developed in patients with MetS than in patients without MetS. The occurrence of risk progression was 1.76 vs 1.52% in low-risk, 16.48 vs 10.87% in intermediate-risk, and 26.25 vs 20.16% in high-risk for MetS vs without MetS patients (all *P* < 0.001).

### Comparison of Survival Rate Between PCa Patients with MetS and Without MetS

**Fig. 4:**
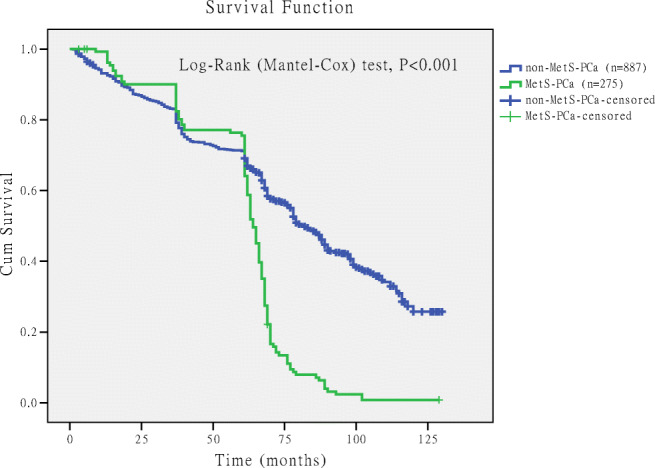
Comparison of the unadjusted survival curves in prostate cancer patients with metabolic syndrome (MetS-PCa) and without metabolic syndrome (non-MetS-PCa). The unadjusted survival curve drawn by Kaplan-Meier method, and “+” represents the censored data of death on the curve. The median survival time of MetS-PCa was 64 months, and the non-MetS-PCa was 82 months. Log-rank (Mantel-Cox) test was used for comparison, and the difference was statistically significant (chi-square value = 82.586, *P* < 0.001)

Figure 4 shows survival rate of MetS-PCa and non-MetS-PCa patients, and the survival curve was drawn using Kaplan-Meier method. The median survival time of MetS-PCa was 64 months, and the non-MetS-PCa was 82 months. Log-rank (Mantel-Cox) test was used for comparison, and the difference was statistically significant [chi-square value(*χ*^2^) = 82.586, *P* < 0.001].

**Fig. 5:**
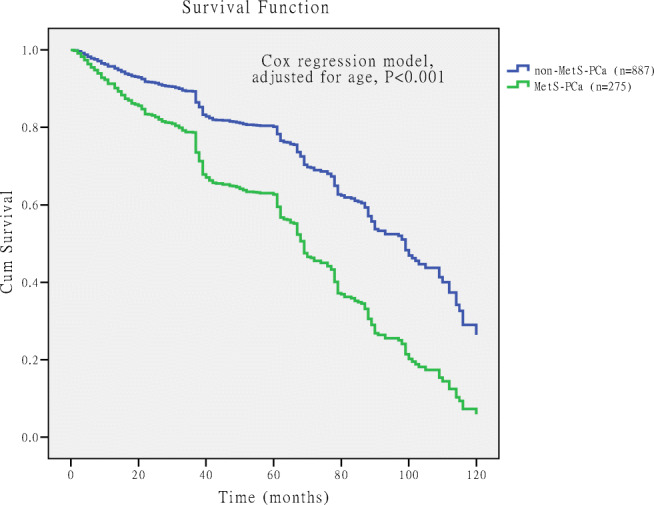
Comparison of the adjusted survival curve survival rate for prostate cancer patients with metabolic syndrome (MetS-PCa) and without metabolic syndrome (non-MetS-PCa). Age included in the Cox regression model, and after adjusting for age, the survival rate of MetS-PCa was lower than non-MetS-PCa, and the difference was statistically significant (*P* < 0.001)

The survival curve was crossed, indicating that the confounding factors affected the survival time; we considered age as the main confounding factor affecting the survival time (Fig. 4). It was not appropriate to use the log-rank test for analysis, and analysis should be carried out using Cox regression model. We found that both age (RR = 1.065, 95%CI 1.054–1.076, *P* < 0.001) and MetS (RR = 3.473, 95%CI 3.382–3.585, *P* < 0.001) were the risk factors associated with the survival time of PCa after performing the Cox regression model. After adjustment for age, the survival curves of MetS were redrawn (Fig. 5). The results revealed that the survival rate of MetS-PCa was lower than non-MetS-PCa, and the difference was statistically significant (*P* < 0.001).

### The Differences of CRP and PDW in MetS-PCa Patients with Non-MetS-PCa Patients

The two indicators CPR and PDW were accompanied with chronic inflammatory state and were higher in patients with MetS than without MetS. The median level of CRP and PDW was 8.6(6.6~12.4) vs 5.7(3.9~8.2) mg/l (*P* = 0.002) and 17.2% (12.6~21.6%) vs 13.6% (11.4~16.8%) (*P* = 0.005) in the MetS vs without MetS patients. Multivariate logistic regression analysis showed independent association of CRP (OR = 1.077, 95%CI 1.011–1.148, *P* = 0.022) and PDW (OR = 1.202, 95%CI 1.112–1.298, *P* < 0.001) with treatment.

## Discussion

In this study, we analyzed the relationship between PCa and MetS and the effect of PCa on MetS patients after undergoing various treatments. Our present study results showed that the occurrence of MetS was influenced by many factors, such as age, poor diet and lack of exercise, progression of PCa itself, as well as a variety of treatments in prostate cancer patients. We focused on a variety of PCa treatment effects on MetS and according to the effect of each kind of treatment on the body metabolism, we subjectively classified the treatment groups and found that PCa treatment with ADT+^125^I limited+chemotherapy led to MetS. However, the effect of RP+TURP+EBRT treatment on MetS was relatively small. These findings help us choose a more appropriate treatment for PCa, and avoid MetS side effects as much as possible.

MetS is one of the most common comorbidity seen in a majority of patients with PCa during the course of development process. Many treatment options have been used clinically for the treatment of PCa. Despite their beneficial effects on survival, various therapeutic methods, especially ADT, may also induce numerous adverse effects that overlap with MetS and remains poorly understood [3, 6, 13, 14, 16, 19, 20]. This study using retrospective data in multicenter PCa patients found that different treatment options for PCa may have an impact on MetS. As shown in Table 1 and Fig. 1, a difference in the incidence of MetS in PCa patients existed before and after treatments, and treatment options were regarded as independent risk factors for MetS development (OR = 1.731, 95%CI 1.367–2.193, *P* < 0.001) by ruling out the influence of mixed factors including age, unhealthy living habits (like smoking, drinking, high-carb high-fat diet, and lack of enough sports), dwelling environment, education background, PSA, Gleason score, and clinical stage. Further analysis of the effects on MetS by various treatment methods for PCa (Table 3) demonstrated that the incidence of MetS after various treatments for PCa were different. The increased MetS incidence was found after ^125^I limited, MC, and chemotherapy; decreased MetS incidence was found after RP and SC; and the rates of MetS incidence remained unchanged after EBRT and TURP treatments. Multivariate logistic regression analysis showed that ^125^I limited, MC, and chemotherapy were independent risk factors for MetS, but RP and SC were protective factors for MetS. Furthermore, the change of MetS component abnormalities was more obvious after PCa treatments (Fig. 1). The total proportion of subjects with one or more component abnormality of MetS was higher after treatment than before treatment. Thus, analysis of our data showed that different treatment strategies for PCa may determine the occurrence, development, and transition of MetS clinically. What is more, the possible reason for this may be that the various treatment strategies for PCa differ in timing and intensity of the body’s metabolic interference.

Previous literature reported that the prevalence of MetS was 16–18.36% in Chinese older men (65 to 80 years) in non-PCa patients [21, 22], and the incidence of PCa with MetS (MetS-PCa) was 11% and PCa without MetS (non-MetS-PCa) was 13% in the age range of 70–80 years [23]. In this study, the prevalence of MetS before (17.38%) and after (23.67%) PCa treatment showed significant differences (*P* < 0.001; see Table 1), and the total proportion of subjects with one or more component abnormality of MetS was higher in the treated patients than in patients before treatment (80.08 vs 55.42%, *P* < 0.001; see Fig. 1). We found that the increasing age as an independent risk factor for the onset of MetS, and this was consistent with the previous literature reports [21–23]. In a word, in this study, differences existed in the incidence of MetS before and after treatments, and the general trend was that the incidence of MetS posttreatment was higher than before treatment, which was independently associated with various treatments (OR = 1.731, 95%CI 1.367–2.193, *P* < 0.001) through multivariate logistic regression analysis (see Table 1) after adjusting for confounding factors such as age (OR = 1.042, 95%CI 1.028–1.056, *P* < 0.001).

Considering different effects on the systematic metabolism by various treatment methods, we further divided the treatments into two categories subjectively to understand their influence on MetS: RP+TURP+EBRT and ADT (including surgical or medical castration)+^125^I limited+chemotherapy. Meanwhile, to minimize the impact of tumor staging and grading on the development of MetS, the subjects were evaluated by different risk grades of PCa (low-risk, intermediate-risk, and high-risk) according to the NCCN criteria [17]. This helps us for the better management of the disease [24]. As shown in Fig. 2, the newly developed MetS patients were different between the two treatment groups in all the risk grades of PCa. In total, 228 PCa patients had newly developed MetS, and MetS incidence was higher in ADT+^125^I limited+chemotherapy group than in RP+TURP+EBRT group of any risk grades. Through multivariate logistic regression analysis, MetS was associated with advanced PSA increase, recurrence, and metastasis, except for death (see Table 2). Meanwhile, as shown in Fig. 3, clinical progression of PCa after treatments showed higher proportion in patients with MetS than without MetS in the corresponding risk factor group. The above discussed results showed that MetS was an independent risk factor for the clinical progression of PCa. These results implied that the influence of ADT, ^125^I limited, and chemotherapy on MetS may be more pronounced, with the increased incidence of MetS during the course of the treatment process and may have an impact on the PCa progression. Such discrepancy may explain the differences between our results and those of the previous research studies. Nevertheless, results from this study might have supplementary values for previous studies [6].

In addition, our data showed that the median scores of storage symptoms and voiding symptoms were higher in the MetS group than the non-MetS group, and the development of voiding symptoms demonstrated urinary obstruction as more serious than that of storage symptoms under the impact of MetS. Multivariate logistic regression analysis further showed that high lower urinary tract symptom score was an independent risk factor for MetS (see Table 1). These study findings supported the results of previous research studies [25, 26].

Our data also showed that the therapeutic outcome had an impact on PCa patients combined with MetS. Survival analysis showed that the median survival time of MetS-PCa was 64 months, and the non-MetS-PCa was 82 months (see Fig. 4). Cox regression model revealed that both age and MetS were the risk factors for the survival time of PCa, and the relative risk (RR) was 1.065 for age and 3.473 for MetS. After adjustment for age, the survival curves of MetS were redrawn (see Fig. 5) and found that the survival rate of MetS-PCa was lower than non-MetS-PCa, and the difference was statistically significant (*P* < 0.001). So, the prognosis of MetS-PCa patients was worse than those of non-MetS-PCa patients. Results of this study are different from the previous studies, and moreover, it was a useful complement for the previous research findings [9, 27]. Interestingly, the mortality (see Table 2) of PCa patients does not correlate with MetS. The reason for this might be that the patients ultimately died due to cardiovascular complications or multiple organ failure [9, 27].

MetS is normally associated with a chronic inflammatory state, playing an important role in the development of MetS and advancement of malignant tumors [8, 9, 25, 28]. In this study, total subjects of PCa were divided into patients with MetS and without MetS to analyze the differences of PDW and CRP. The two inflammatory markers were significantly higher in MetS group than without MetS group. Multivariate logistic regression analysis showed that PDW and CRP were positively and independently correlated with MetS after adjusting for various mixed factors. These results are similar to the previous study findings, where chronic inflammation and inflammatory markers may have a role in PCa patients with MetS [8, 9, 25, 28]. So, it may be necessary to consider the inflammatory markers, such as CRP and PDW, when exploring for the possible treatments in PCa patients [9, 29–32] and eliminating or inhibiting chronic inflammation may be one of the supplementary methods for the treatment of PCa combined with MetS.

However, the present study has some limitations. This study was conducted only in Chinese population, which included majority of Han Chinese, and also contained a small proportion of Hui, Miao, Uighur, and Tibetan inhabitants who have different eating habits, but our study did not subdivide the participants. Our study was based on only two clinical medical centers and with relatively few cases, especially in the partial treatment groups, such as EBRT and TURP. These limitations may influence our judgment of the actual results and conclusions. Hence, more centralized and large case studies are needed.

In summary, we have to admit that multiple factors among PCa patients contribute to the occurrence of MetS. The treatment group of ADT+^125^I limited+chemotherapy may be the most influential factor with MetS, but the effect of RP+TURP+EBRT treatment on MetS was relatively small. These findings can help us choose a more appropriate treatment strategy for PCa, and avoid side effects of MetS as much as possible. MetS-PCa patients generally facilitate to cancer progression, and chronic inflammation may have a role in MetS-PCa patients.
